# 1-Phenyl-5-{[2-(trimethyl­sil­yl)eth­yl]sulfon­yl}-1*H*-tetra­zole

**DOI:** 10.1107/S1600536811030492

**Published:** 2011-08-17

**Authors:** David Tymann, Björn Nelson, Carsten Strohmann, Hans Preut, Martin Hiersemann

**Affiliations:** a Fakultät Chemie, Technische Universität Dortmund, Otto-Hahn-Strasse 6, 44221 Dortmund, Germany

## Abstract

The title compound, C_12_H_18_N_4_O_2_SSi, was synthesized to be employed in a Julia–Kocieński olefination. In the mol­ecule, the dihedral angle between the phenyl ring and the tetra­zole ring is 41.50 (5)°. The significantly longer Si—C(methyl­ene) bond [1.8786 (13) Å] and the shortened adjacent C—C bond [1.5172 (18) Å], as well as the significant deviation of the corresponding Si—C—C angle [114.16 (9)°] from the ideal tetra­hedral angle, can be attributed to the β-effect of silicon. In the crystal, mol­ecules are held together by van der Waals inter­actions.

## Related literature

For Julia–Kocieński olefination, see: Blakemore *et al.* (1998 ▶). For the use of unsaturated α-keto esters in intra­molecular carbonyl-ene reactions in natural product synthesis, see: Helmboldt & Hiersemann (2009 ▶); Helmboldt *et al.* (2006 ▶); Schnabel & Hiersemann (2009 ▶); Schnabel *et al.* (2011 ▶) The title compound was synthesized using a reduction of ethyl 2-(trimethyl­sil­yl)acetate (Gerlach, 1977 ▶) followed by a Mitsunobu reaction (Mitsunobu & Yamada, 1967 ▶; Mitsunobu *et al.*, 1967 ▶) and a subsequent Mo-(VI)-catalyzed oxidation of the thio­ether (Schultz *et al.*, 1963 ▶). 
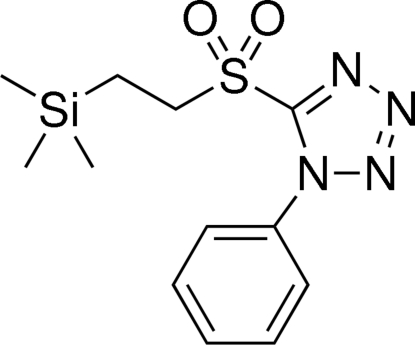


## Experimental

### 

#### Crystal data

C_12_H_18_N_4_O_2_SSi
*M* *_r_* = 310.45
Monoclinic, 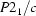

*a* = 11.3126 (4) Å
*b* = 13.2707 (4) Å
*c* = 10.8277 (4) Å
β = 106.902 (4)°
*V* = 1555.31 (9) Å^3^
*Z* = 4
Mo *K*α radiation
μ = 0.29 mm^−1^
*T* = 173 K
0.50 × 0.50 × 0.20 mm

#### Data collection

Oxford Diffraction Xcalibur S CCD diffractometer
Absorption correction: multi-scan (*CrysAlis RED*; Oxford Diffraction, 2008 ▶) *T*_min_ = 0.868, *T*_max_ = 0.944
22841 measured reflections
3384 independent reflections
2961 reflections with *I* > 2σ(*I*)
*R*_int_ = 0.024

#### Refinement

*R*[*F* ^2^ > 2σ(*F* ^2^)] = 0.027
*wR*(*F* ^2^) = 0.079
*S* = 1.10
3384 reflections
184 parameters
H-atom parameters constrained
Δρ_max_ = 0.37 e Å^−3^
Δρ_min_ = −0.37 e Å^−3^

### 

Data collection: *CrysAlis CCD* (Oxford Diffraction, 2008 ▶); cell refinement: *CrysAlis CCD*; data reduction: *CrysAlis CCD*; program(s) used to solve structure: *SHELXS97* (Sheldrick, 2008 ▶); program(s) used to refine structure: *SHELXL97* (Sheldrick, 2008 ▶); molecular graphics: *SHELXTL-Plus* (Sheldrick, 2008 ▶); software used to prepare material for publication: *SHELXL97* and *PLATON* (Spek, 2009 ▶).

## Supplementary Material

Crystal structure: contains datablock(s) I, global. DOI: 10.1107/S1600536811030492/hg5066sup1.cif

Structure factors: contains datablock(s) I. DOI: 10.1107/S1600536811030492/hg5066Isup2.hkl

Supplementary material file. DOI: 10.1107/S1600536811030492/hg5066Isup3.cml

Additional supplementary materials:  crystallographic information; 3D view; checkCIF report

## Figures and Tables

**Fig. 1.**
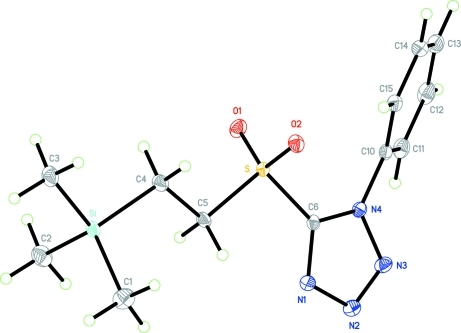
: The molecular structure of the title compound, showing the labelling of all non-H atoms. Displacement ellipsoids are shown at the 30% probability level.

## supplementary crystallographic information

### Comment

On the search for an alternative synthetic route for the preparation of unsaturated α-keto esters we envisioned the Julia–Kocieński olefination (Blakemore *et al.*, 1998) as a key connecting step for the construction of the C=C-double bond. Unsaturated α-keto esters have been successfully employed in intramolecular carbonyl-ene reactions in natural product synthesis (Helmboldt *et al.*,  2006; Helmboldt & Hiersemann, 2009; Schnabel & Hiersemann, 2009; Schnabel *et al.*, 2011). For the preparation of the title compound I, 1-phenyl-5-((2-(trimethylsilyl)ethyl)thio)-1*H*-tetrazole was synthesized using a reduction of ethyl 2-(trimethylsilyl)acetate (Gerlach, 1977) followed by a Mitsunobu reaction (Mitsunobu & Yamada, 1967; Mitsunobu *et al.*, 1967). A subsequent Mo-(VI)-catalyzed oxidation of the thioether II (Schultz  *et al.*, 1963) gave the title compound (I).

### Experimental

To a solution of II (6.07 g, 21.8 mmol, 1.0 eq) in ethanol (220 ml, 10 ml/mmol II) was added a solution of (NH_4_)Mo_7_O_24_.2H_2_O (2.70 g, 0.22 mmol, 0.1 eq) in 35% aqueous H_2_O_2_ (18.75 ml, 0.22 mol, 10.0 eq). After stirring at room temperature for 22 h the reaction mixture was diluted with aqueous NH_4_Cl solution and methylene chloride. The layers were separated and the aqueous phase was extracted with methylene chloride (3x). The combined organic phases were washed with water (3x), dried over anhydrous MgSO_4_, filtered, and concentrated under reduced pressure (323 K, 0.05 mbar) to afford I (6.72 g, 21.7 mmol, 99%) as crystals. Single crystals of I were obtained by recrystallization from *n*-pentane to give colorless cuboids: *R*_f_ 0.63 (cyclohexane/ethyl acetate 5/1); ^1^H NMR (CDCl_3_, 400 MHz, δ): 0.11 (s, 9H), 1.10–1.16 (m, 2H), 3.64–3.70 (m, 2H), 7.61–7.70 (m, 5H); ^13^C NMR (CDCl_3_, 101 MHz, δ): -1.8 (3xCH_3_), 8.4 (CH_2_), 53.3 (CH_2_), 125.3 (2xCH), 129.9 (2xCH), 131.7 (CH), 133.3 (C), 153.5 (C); IR (cm^-1^): 2955 (w) (ν_as,s_ C—H, CH_3_, CH_2_), 1496 (*m*) (ν C=C, Ar), 1426 (w), 1347 (*s*) (ν*R*_2_SO_2_), 1251 (*m*), 1172 (*m*), 1152 (*s*), 1111 (w), 1014 (w), 834 (*m*); Anal. Calcd. for C_12_H_18_N_4_O_2_SSi: C, 46.4; H, 5.8; N, 18.1; Found: C, 46.4; H, 5.8; N, 17.9; *M* = 310.45 g/mol.

### Refinement

All H  atoms were placed at idealised positions and refined as riding [C-H = 0.95 Å (aromatic C), 0.99 Å (CH_2_ and 0.98 Å (CH_3_), U_iso_(H) = 1.2 U_eq_(C) (aromatic and CH_2_) and 1.5 U_eq_(C) (CH_3_)].

### Crystal data

**Table:**

C_12_H_18_N_4_O_2_SSi	*F*(000) = 656
*M**_r_* = 310.45	*D*_x_ = 1.326 Mg m^−^^3^
Monoclinic, *P*2_1_/*c*	Mo *K*α radiation, λ = 0.71073 Å
Hall symbol: -P 2ybc	Cell parameters from 16653 reflections
*a* = 11.3126 (4) Å	θ = 2.4–29.2°
*b* = 13.2707 (4) Å	µ = 0.29 mm^−^^1^
*c* = 10.8277 (4) Å	*T* = 173 K
β = 106.902 (4)°	Block, colourless
*V* = 1555.31 (9) Å^3^	0.50 × 0.50 × 0.20 mm
*Z* = 4	

### Data collection

**Table:**

Oxford Diffraction Xcalibur S CCD diffractometer	3384 independent reflections
Radiation source: Enhance (Mo) X-ray Source	2961 reflections with *I* > 2σ(*I*)
graphite	*R*_int_ = 0.024
Detector resolution: 16.0560 pixels mm^-1^	θ_max_ = 27.0°, θ_min_ = 2.4°
ω scans	*h* = −14→14
Absorption correction: multi-scan (*CrysAlis RED*; Oxford Diffraction, 2008)	*k* = −16→16
*T*_min_ = 0.868, *T*_max_ = 0.944	*l* = −13→13
22841 measured reflections	

### Refinement

**Table:**

Refinement on *F*^2^	Primary atom site location: structure-invariant direct methods
Least-squares matrix: full	Secondary atom site location: difference Fourier map
*R*[*F*^2^ > 2σ(*F*^2^)] = 0.027	Hydrogen site location: inferred from neighbouring sites
*wR*(*F*^2^) = 0.079	H-atom parameters constrained
*S* = 1.10	*w* = 1/[σ^2^(*F*_o_^2^) + (0.0465*P*)^2^ + 0.3668*P*] where *P* = (*F*_o_^2^ + 2*F*_c_^2^)/3
3384 reflections	(Δ/σ)_max_ = 0.001
184 parameters	Δρ_max_ = 0.37 e Å^−^^3^
0 restraints	Δρ_min_ = −0.37 e Å^−^^3^

### Special details

**Table:**

Experimental. CrysAlis RED, Oxford Diffraction Ltd., Version 1.171.32.37 (release 24-10-2008) Empirical absorption correction using sperical harmonics, implemented in SCALE3 ABSPACK scaling algorithm.
Geometry. All e.s.d.'s (except the e.s.d. in the dihedral angle between two l.s. planes) are estimated using the full covariance matrix. The cell e.s.d.'s are taken into account individually in the estimation of e.s.d.'s in distances, angles and torsion angles; correlations between e.s.d.'s in cell parameters are only used when they are defined by crystal symmetry. An approximate (isotropic) treatment of cell e.s.d.'s is used for estimating e.s.d.'s involving l.s. planes.
Refinement. Refinement of *F*^2^ against ALL reflections. The weighted *R*-factor *wR* and goodness of fit *S* are based on *F*^2^, conventional *R*-factors *R* are based on *F*, with *F* set to zero for negative *F*^2^. The threshold expression of *F*^2^ > σ(*F*^2^) is used only for calculating *R*-factors(gt) *etc*. and is not relevant to the choice of reflections for refinement. *R*-factors based on *F*^2^ are statistically about twice as large as those based on *F*, and *R*- factors based on ALL data will be even larger.

### Fractional atomic coordinates and isotropic or equivalent isotropic displacement parameters (Å^2^)

**Table:**

	*x*	*y*	*z*	*U*_iso_*/*U*_eq_	
Si	0.47993 (3)	0.79710 (3)	1.25192 (3)	0.01949 (10)	
C1	0.47042 (15)	0.87175 (12)	1.39384 (14)	0.0341 (3)	
H1A	0.3933	0.8557	1.4135	0.051*	
H1B	0.4722	0.9438	1.3744	0.051*	
H1C	0.5408	0.8552	1.4684	0.051*	
C2	0.61720 (14)	0.83876 (13)	1.20403 (15)	0.0346 (3)	
H2A	0.6923	0.8247	1.2743	0.052*	
H2B	0.6114	0.9113	1.1862	0.052*	
H2C	0.6203	0.8022	1.1263	0.052*	
C3	0.48654 (13)	0.66005 (11)	1.28620 (15)	0.0310 (3)	
H3A	0.4895	0.6228	1.2090	0.046*	
H3B	0.4130	0.6399	1.3105	0.046*	
H3C	0.5607	0.6450	1.3573	0.046*	
C4	0.33521 (12)	0.81809 (10)	1.11572 (13)	0.0255 (3)	
H4A	0.3418	0.7802	1.0393	0.031*	
H4B	0.2643	0.7903	1.1407	0.031*	
C5	0.30914 (11)	0.92782 (10)	1.07831 (12)	0.0206 (3)	
H5A	0.3797	0.9573	1.0541	0.025*	
H5B	0.2977	0.9664	1.1523	0.025*	
S	0.17351 (3)	0.93484 (2)	0.94605 (3)	0.01563 (9)	
O1	0.19901 (9)	0.90232 (7)	0.83074 (9)	0.0253 (2)	
O2	0.07296 (8)	0.89053 (7)	0.98167 (9)	0.0222 (2)	
C6	0.14770 (11)	1.06672 (9)	0.93227 (12)	0.0162 (2)	
N1	0.20780 (10)	1.12950 (8)	1.02188 (10)	0.0209 (2)	
N2	0.16789 (11)	1.22251 (8)	0.97617 (11)	0.0253 (3)	
N3	0.08670 (11)	1.21661 (8)	0.86430 (11)	0.0233 (2)	
N4	0.07262 (9)	1.11767 (8)	0.83393 (10)	0.0172 (2)	
C10	−0.01458 (11)	1.08607 (10)	0.71540 (12)	0.0187 (3)	
C11	−0.02014 (13)	1.14131 (11)	0.60586 (13)	0.0254 (3)	
H11	0.0318	1.1982	0.6096	0.030*	
C12	−0.10312 (14)	1.11189 (12)	0.49061 (13)	0.0320 (3)	
H12	−0.1085	1.1485	0.4138	0.038*	
C13	−0.17799 (14)	1.02960 (12)	0.48703 (14)	0.0331 (3)	
H13	−0.2349	1.0098	0.4075	0.040*	
C14	−0.17131 (13)	0.97537 (11)	0.59818 (14)	0.0295 (3)	
H14	−0.2235	0.9187	0.5945	0.035*	
C15	−0.08875 (12)	1.00359 (10)	0.71465 (13)	0.0223 (3)	
H15	−0.0834	0.9672	0.7916	0.027*	

### Atomic displacement parameters (Å^2^)

**Table:**

	*U*^11^	*U*^22^	*U*^33^	*U*^12^	*U*^13^	*U*^23^
Si	0.01680 (18)	0.01994 (18)	0.02019 (18)	0.00148 (13)	0.00294 (13)	0.00318 (13)
C1	0.0433 (9)	0.0335 (8)	0.0261 (7)	0.0030 (7)	0.0110 (6)	0.0011 (6)
C2	0.0267 (7)	0.0423 (9)	0.0372 (8)	−0.0053 (7)	0.0130 (6)	−0.0001 (7)
C3	0.0248 (7)	0.0253 (7)	0.0384 (8)	0.0043 (6)	0.0022 (6)	0.0080 (6)
C4	0.0224 (6)	0.0195 (6)	0.0288 (7)	0.0011 (5)	−0.0019 (5)	0.0030 (5)
C5	0.0172 (6)	0.0206 (6)	0.0198 (6)	0.0009 (5)	−0.0012 (5)	0.0015 (5)
S	0.01427 (15)	0.01592 (15)	0.01611 (15)	0.00142 (11)	0.00350 (11)	0.00039 (10)
O1	0.0287 (5)	0.0278 (5)	0.0199 (5)	0.0066 (4)	0.0081 (4)	−0.0022 (4)
O2	0.0181 (4)	0.0204 (5)	0.0283 (5)	−0.0012 (4)	0.0069 (4)	0.0039 (4)
C6	0.0142 (5)	0.0170 (6)	0.0184 (6)	0.0009 (4)	0.0064 (4)	0.0012 (4)
N1	0.0185 (5)	0.0195 (5)	0.0242 (5)	−0.0010 (4)	0.0055 (4)	−0.0021 (4)
N2	0.0255 (6)	0.0192 (6)	0.0300 (6)	−0.0004 (4)	0.0064 (5)	−0.0022 (5)
N3	0.0266 (6)	0.0157 (5)	0.0278 (6)	−0.0007 (4)	0.0081 (5)	−0.0010 (4)
N4	0.0188 (5)	0.0152 (5)	0.0182 (5)	0.0011 (4)	0.0064 (4)	0.0017 (4)
C10	0.0176 (6)	0.0202 (6)	0.0174 (6)	0.0052 (5)	0.0039 (5)	−0.0006 (5)
C11	0.0289 (7)	0.0250 (7)	0.0233 (6)	0.0073 (6)	0.0092 (5)	0.0056 (5)
C12	0.0391 (8)	0.0355 (8)	0.0196 (7)	0.0160 (7)	0.0058 (6)	0.0050 (6)
C13	0.0326 (8)	0.0369 (8)	0.0223 (7)	0.0143 (7)	−0.0040 (6)	−0.0076 (6)
C14	0.0250 (7)	0.0250 (7)	0.0331 (8)	0.0032 (6)	−0.0004 (6)	−0.0061 (6)
C15	0.0218 (6)	0.0212 (6)	0.0224 (6)	0.0034 (5)	0.0039 (5)	0.0008 (5)

### Geometric parameters (Å, °)

**Table:**

Si—C3	1.8534 (15)	S—O2	1.4295 (9)
Si—C1	1.8575 (15)	S—C6	1.7734 (13)
Si—C2	1.8586 (15)	C6—N1	1.3093 (16)
Si—C4	1.8786 (13)	C6—N4	1.3366 (15)
C1—H1A	0.9800	N1—N2	1.3573 (16)
C1—H1B	0.9800	N2—N3	1.2924 (16)
C1—H1C	0.9800	N3—N4	1.3517 (15)
C2—H2A	0.9800	N4—C10	1.4353 (15)
C2—H2B	0.9800	C10—C15	1.3778 (19)
C2—H2C	0.9800	C10—C11	1.3802 (18)
C3—H3A	0.9800	C11—C12	1.382 (2)
C3—H3B	0.9800	C11—H11	0.9500
C3—H3C	0.9800	C12—C13	1.376 (2)
C4—C5	1.5172 (18)	C12—H12	0.9500
C4—H4A	0.9900	C13—C14	1.385 (2)
C4—H4B	0.9900	C13—H13	0.9500
C5—S	1.7710 (12)	C14—C15	1.3850 (18)
C5—H5A	0.9900	C14—H14	0.9500
C5—H5B	0.9900	C15—H15	0.9500
S—O1	1.4275 (9)		
			
C3—Si—C1	111.49 (7)	H5A—C5—H5B	108.3
C3—Si—C2	111.06 (7)	O1—S—O2	119.38 (6)
C1—Si—C2	109.04 (8)	O1—S—C5	110.09 (6)
C3—Si—C4	106.14 (6)	O2—S—C5	109.30 (6)
C1—Si—C4	108.88 (7)	O1—S—C6	107.15 (6)
C2—Si—C4	110.18 (7)	O2—S—C6	107.71 (6)
Si—C1—H1A	109.5	C5—S—C6	101.69 (6)
Si—C1—H1B	109.5	N1—C6—N4	109.95 (11)
H1A—C1—H1B	109.5	N1—C6—S	121.89 (9)
Si—C1—H1C	109.5	N4—C6—S	128.11 (9)
H1A—C1—H1C	109.5	C6—N1—N2	105.22 (10)
H1B—C1—H1C	109.5	N3—N2—N1	110.92 (10)
Si—C2—H2A	109.5	N2—N3—N4	106.75 (10)
Si—C2—H2B	109.5	C6—N4—N3	107.16 (10)
H2A—C2—H2B	109.5	C6—N4—C10	132.60 (11)
Si—C2—H2C	109.5	N3—N4—C10	120.20 (10)
H2A—C2—H2C	109.5	C15—C10—C11	122.78 (12)
H2B—C2—H2C	109.5	C15—C10—N4	119.77 (11)
Si—C3—H3A	109.5	C11—C10—N4	117.45 (12)
Si—C3—H3B	109.5	C10—C11—C12	118.37 (14)
H3A—C3—H3B	109.5	C10—C11—H11	120.8
Si—C3—H3C	109.5	C12—C11—H11	120.8
H3A—C3—H3C	109.5	C13—C12—C11	120.05 (13)
H3B—C3—H3C	109.5	C13—C12—H12	120.0
C5—C4—Si	114.16 (9)	C11—C12—H12	120.0
C5—C4—H4A	108.7	C12—C13—C14	120.69 (13)
Si—C4—H4A	108.7	C12—C13—H13	119.7
C5—C4—H4B	108.7	C14—C13—H13	119.7
Si—C4—H4B	108.7	C15—C14—C13	120.17 (14)
H4A—C4—H4B	107.6	C15—C14—H14	119.9
C4—C5—S	108.77 (9)	C13—C14—H14	119.9
C4—C5—H5A	109.9	C10—C15—C14	117.94 (13)
S—C5—H5A	109.9	C10—C15—H15	121.0
C4—C5—H5B	109.9	C14—C15—H15	121.0
S—C5—H5B	109.9		
			
C3—Si—C4—C5	176.60 (11)	S—C6—N4—N3	−177.75 (9)
C1—Si—C4—C5	56.46 (12)	N1—C6—N4—C10	−177.91 (11)
C2—Si—C4—C5	−63.07 (13)	S—C6—N4—C10	4.50 (19)
Si—C4—C5—S	178.07 (7)	N2—N3—N4—C6	0.40 (14)
C4—C5—S—O1	−74.32 (11)	N2—N3—N4—C10	178.48 (10)
C4—C5—S—O2	58.65 (11)	C6—N4—C10—C15	40.14 (19)
C4—C5—S—C6	172.32 (9)	N3—N4—C10—C15	−137.36 (12)
O1—S—C6—N1	−127.27 (10)	C6—N4—C10—C11	−140.07 (13)
O2—S—C6—N1	103.12 (11)	N3—N4—C10—C11	42.42 (16)
C5—S—C6—N1	−11.74 (12)	C15—C10—C11—C12	−0.5 (2)
O1—S—C6—N4	50.06 (12)	N4—C10—C11—C12	179.73 (12)
O2—S—C6—N4	−79.55 (12)	C10—C11—C12—C13	0.3 (2)
C5—S—C6—N4	165.59 (11)	C11—C12—C13—C14	0.0 (2)
N4—C6—N1—N2	−0.12 (13)	C12—C13—C14—C15	0.0 (2)
S—C6—N1—N2	177.64 (9)	C11—C10—C15—C14	0.5 (2)
C6—N1—N2—N3	0.38 (14)	N4—C10—C15—C14	−179.75 (11)
N1—N2—N3—N4	−0.49 (14)	C13—C14—C15—C10	−0.2 (2)
N1—C6—N4—N3	−0.17 (14)		

## References

[bb1] Blakemore, P. R., Cole, W. J., Morley, A. & Kocieński, P. J. (1998). *Synlett*, pp. 26–28.

[bb2] Gerlach, H. (1977). *Helv. Chim. Acta*, **60**, 3039–3044.

[bb3] Helmboldt, H. & Hiersemann, M. (2009). *J. Org. Chem.* **74**, 1698–1708. 10.1021/jo802581g 19140728

[bb4] Helmboldt, H., Köhler, D. & Hiersemann, M. (2006). *Org. Lett.* **8**, 1573–1576. 10.1021/ol060115t 16597113

[bb5] Mitsunobu, O. & Yamada, M. (1967). *Bull. Chem. Soc. Jpn*, **40**, 2380–2382.

[bb6] Mitsunobu, O., Yamada, M. & Mukaiyama, T. (1967). *Bull. Chem. Soc. Jpn*, **40**, 935–939.

[bb7] Oxford Diffraction (2008). *CrysAlis CCD* and *CrysAlis RED* Oxford Diffraction Ltd, Yarnton, England.

[bb8] Schnabel, C. & Hiersemann, M. (2009). *Org. Lett.* **11**, 2555–2558. 10.1021/ol900819u 19453178

[bb9] Schnabel, C., Sterz, K., Müller, H., Rehbein, J., Wiese, M. & Hiersemann, M. (2011). *J. Org. Chem.* **76**, 512–522. 10.1021/jo1019738 21192665

[bb10] Schultz, H. S., Freyermuth, H. B. & Buc, S. R. (1963). *J. Org. Chem.* **28**, 1140–1142.

[bb11] Sheldrick, G. M. (2008). *Acta Cryst.* A**64**, 112–122. 10.1107/S0108767307043930 18156677

[bb12] Spek, A. L. (2009). *Acta Cryst.* D**65**, 148–155. 10.1107/S090744490804362X PMC2631630 19171970

